# Moral disengagement, self-control and callous-unemotional traits as predictors of cyberbullying: a moderated mediation model

**DOI:** 10.1186/s40359-023-01287-z

**Published:** 2023-08-25

**Authors:** Haojian Li, Qi Guo, Ping Hu

**Affiliations:** 1 School of Social & Behavioral Sciences, Arizona State University, Glendale, AZ United States of America; 2 Department of Psychology, Renmin University of China, Beijing, Haidian district People’s Republic of China

**Keywords:** Cyberbullying, Moral disengagement, Self-control, Callous-unemotional traits, Social media

## Abstract

**Background:**

Cyberbullying has become more prevalent, more difficult to detect, and more harmful to the victims. Whereas considerable prior work has investigated predictors and consequences of cyberbullying, additional research is needed to better understand the mechanisms by which these factors relate to cyberbullying perpetration and victimization. The goal of the present study was to examine the extent to which the link between individual differences in moral disengagement and cyberbullying perpetration is mediated by low self-control and, if so, whether this mediation effect varies by individuals’ degree of callous-unemotional traits.

**Method:**

To explore these questions, we used cyberbullying, moral disengagement, self-control, and callous-unemotional traits scales and collected online survey data from a sample of 860 Chinese internet users aged 18 years old or older.

**Result:**

As hypothesized, a significant positive relation between moral disengagement and cyberbullying emerged that was mediated by individual differences in self-control. Additionally, evidence of moderated mediation was found. That is, the indirect effect varied by degree of callous-unemotional traits, with a significantly stronger mediation effect (and association between self-control and cyberbullying) for individuals who were relatively higher in callous-unemotional traits.

**Conclusion:**

We conclude that moral disengagement partially predicts cyberbullying through self-control, while callous-unemotional traits moderate the pathway between self-control and cyberbullying.

## Background

### Cyberbullying

With the increased use of social media, mobile devices, and online communication, cyberbullying—“using information and communication technologies to repeatedly and intentionally harm, harass, hurt, and/or embarrass a target” [1]—has become more prevalent, difficult to detect, and harmful to the victims [2]. According to estimates, based on different measurement, the prevalence of cyberbullying victimization among children and adolescents ranges from 14.0–57.5% globally, with 44.5% being reported in China alone [3]. Furthermore, an extensive body of research has highlighted the negative impacts associated with cyberbullying, including poorer physical health, psychological distress [4, 5], and a higher risk of suicide among adolescent victims [6]. The significance of investigating cyberbullying is thus emphasized by these findings.

Much of the existing literature has investigated predictors and consequences of cyberbullying [7, 8]. Individual differences in moral disengagement—cognitive processes whereby people disconnect or disengage, from their moral obligations in ways that allow them to harm others without the guilt of self-condemnation [9]—for example, have been identified as a predictor of cyberbullying perpetration [10]. Yet additional research is needed to develop a better understanding of the mechanisms that link various individual difference predictors—including moral disengagement—to cyberbullying. The present study was thus designed to investigate the extent to which one such mechanism, self-control, mediates the relation between moral disengagement and cyberbullying perpetration. Furthermore, we explored callous-unemotional traits as a moderator of the potential pathway between moral disengagement and cyberbullying through self-control.

### Moral disengagement and cyberbullying

Moral disengagement is a significant factor in explaining why individuals who see themselves as moral can perform seemingly immoral behaviors (e.g., intentionally harming others). This term refers to a range of cognitive mechanisms [10] that allow individuals to participate in immoral behaviors while still perceiving consistency with their moral standards. One common form of moral disengagement involves blaming the victims for negative outcomes. If perpetrators can attribute responsibility to the victim, they can maintain a positive self-image as moral individuals [11–13]. This reshapes their self-perception of unethical behavior into acceptable behavior through disengagement mechanisms.

Perhaps not surprisingly, moral disengagement has been linked with increased aggressive behavior, including traditional (face-to-face) bullying. For instance, in a longitudinal study by Sticca and Perren [14], participants with higher levels of moral disengagement reported higher levels of physical and verbal violence. Other research found that adolescents who have bullied others score significantly higher in moral disengagement than victims, non-aggressive adolescents, and those who don’t participate in bullying [15–17].

Recent research has also identified moral disengagement as a predictor of cyberbullying perpetration [10, 18]. It has been proposed that moral disengagement may play an especially strong role in cyberbullying perpetration on social media or online platforms. Because harm to victims may be invisible to perpetrators in online environments, due to the distance or time between the harmful act and the consequent harm, some important cues for the elicitation of empathy in human communication may be eliminated [19]. Relatedly, others have argued that the relative anonymity of the internet provides a social environment that facilitates moral disengagement, and this environmental factor interacts with the individual’s tendencies to promote cyberbullying and immoral behavior online [20].

Consistent with these connected lines of reasoning, Hoareau’s research [21] examined the predictive roles of moral disengagement and psychopathy in cyberbullying and found evidence of a positive relationship between moral disengagement and cyberbullying perpetration. Other research, with samples from Greece and Spain, has yielded similar findings, suggesting a potentially robust relationship between moral disengagement and cyberbullying perpetration across cultures [22–24]. In a research by Hood and Duffy [25], moral disengagement not only corresponded with an increased likelihood of cyberbullying perpetration but also moderated the relation between cyberbullying victimization and perpetration. That is, cyberbullying victimization and perpetration were positively correlated, but this association was significantly stronger among participants with higher levels of moral disengagement.

Interestingly, some researchers have proposed that moral disengagement may factor *less* prominently in cyberbullying perpetration and cyberbystander behavior than in traditional bullying because aggression in the online context may require less justification, obviating the need for a specific cognitive process like moral disengagement [26]. Nevertheless, the literature generally indicates that moral disengagement is positively correlated with cyberbullying perpetration [10].

In summary, moral disengagement can help individuals engage in immoral behavior while alleviating their internal moral burden, maintaining their inner judgment of consistency between their actions and moral standards, and is considered to have a significant relationship with cyberbullying perpetration. However, as will be elaborated in subsequent sections, moral disengagement correlates with other psychological attributes, such as self-control, in predicting aggressive behavior. More research is required to study the mechanism through which moral disengagement impacts cyberbullying. Furthermore, there remains controversy over whether moral disengagement can predict cyberbullying as effectively as it predicts traditional bullying [26].Therefore, further research is needed to investigate this issue.

### Mediating role of self-control

A question that warrants further attention concerns the mechanisms through which moral disengagement relates to cyberbullying perpetration. It may be the case that moral disengagement precedes a reduction in self-control—defined as the effortful capacity of the individual to regulate their emotions, thoughts, impulses, or other well-learned or automatic behavioral responses [27]—thereby reducing attempts to inhibit cyberbullying (and other forms of aggressive) behavior.

In many prior studies, self-control has been identified as one of the key factors in directly predicting aggressive behavior, particularly self-control failure can lead to aggressive behaviors. Previous experimental research presents evidence that self-control failures often predict aggression, and increasing self-control can decrease aggression [28]. Self-control capacity relies on a limited resource that can become temporarily depleted, and provoked individuals behave more aggressively when they are depleted than when they are not [29, 30]. Due to that, self-control failure has been identified as a crucial predictor of aggression toward strangers [31],

Of particular relevance, prior research has established a link between self-control and cyberbullying perpetration. Recent research has shed light on a specific type of self-control failure—social media self-control failure [32]—as a predictor of increased levels of aggression on social media and cyberbullying. In a study of South Korean youth, Stults and You [33] found that low self-control was a significant predictor of cyberbullying and research by Kim [34] found that self-control and delinquent peer associations jointly predicted cyberbullying perpetration. Additionally, other studies have found that self-control has both direct and indirect effects on cyberbullying. A cross-cultural study by Vazsonyi [35], for example, showed that among participants from 25 European countries, low self-control predicted higher levels of traditional bullying and cyberbullying perpetration.

Prior research underscores a close link between moral disengagement and self-control, collectively influencing a wide array of aggressive behaviors. For instance, studies have demonstrated that moral disengagement moderates the relationship between self-control and various aggressive behaviors, including verbal aggression and hostility, concurrently attenuating the effect of self-control on these behaviors [36]. Moreover, self-control has been identified as a moderator in the relationship between moral disengagement and traditional bullying [37]. In research by Alexandra [38], self-control moderated the relation between certain social worldviews (e.g., cynicism about human nature and social institutions, belief in fate) and moral disengagement, such that the relation was particularly strong among those with lower self-control. In a behavioral demonstration of the interrelations among moral disengagement, self-control, and aggression, Gabbiadini and colleagues [39] found that individuals with higher levels of moral disengagement were more likely to show a decrease in self-control after playing a violent (versus non-violent) video game, which indicates the close tie between moral disengagement and self-control in computer-human interaction. Considering the correlation of cyberbullying compared to other types of aggressive behavior, it is reasonable to speculate that the relationship moral disengagement, self-control and aggressive behavior can be equally applied to cyberbullying, despite each of the two variables individually has been demonstrated to predict cyberbullying.

Drawing from previous literature and our findings, it is reasonable to infer that moral disengagement and self-control jointly predict a portion of cyberbullying behavior. However, there are also studies that provide counterexamples to this hypothesis, suggesting that the interaction between moral disengagement and self-control is not significantly related to immoral behaviors like cyberstalking [40]. Thus, further research is required to confirm this hypothesis. Furthermore, given that self-control failure leads to a direct increase in aggression behavior [28] while moral disengagement may potentially exert an influence on both the level of self-control [39] and cyberbullying, it is reasonable to speculate that perhaps moral disengagement predicts cyberbullying behavior to some extent through changes in self-control. Because of that, we hypothesis that self-control mediates the relation between moral disengagement and cyberbullying.

### Callous-unemotional traits and their relationship with self-control and cyberbullying

Additional insights may also be gained by taking individual differences in callous-unemotional traits into account. Callous-unemotional traits are a collection of pathological personality traits related to aggression and antisocial behavior. Individuals with callous-unemotional traits often stand out on at least three dimensions of psychopathy: [1] deficient affective experience; [2] an arrogant and deceitful interpersonal style involving a narcissistic view of oneself and cunning and manipulative behavior; and [3] an impulsive and irresponsible behavioral style involving poorly planned behavior and proneness to boredom [41].

About the relationship between self-control and callous-unemotional traits, a theoretical prediction is based on the dual-system model, which separates the self-control process into three components: the impulse system, the self-control system, and state or trait regulating variables [42]. Individuals with high callous-unemotional traits may tend to make impulsive risk-taking and aggressive behaviors, which is likely to boost the impulsive system of self-control. Additionally, individuals with callous-unemotional traits often lack risk avoidance awareness, lack empathy, and tend to adopt ruthless aggressive behaviors to achieve their goals [24, 43]Such behavior patterns complicate self-control and potentially lead to its failure in inhibiting aggressive behaviors. Consequently, these traits or tendencies may predispose such individuals to engage more readily in unethical or aggressive behavior. In fact, many researchers have already found that callous-unemotional traits were significantly positively associated with cyberbullying perpetration [44–46].

Considering the theoretical relationship between callous-unemotional traits and self-control, and the fact that previous studies have shown in the moderation effect between self-control (self-regulation) and callous-unemotional traits on risk taking behavior [47], a plausible hypothesis is that callous-unemotional traits, in conjunction with self-control, predict cyberbullying perpetration, while also acting as a moderator between self-control and cyberbullying perpetration.

### Current study

In summary, previous research has suggested potential links between moral disengagement, self-control, callous-unemotional traits, and cyberbullying. However, there remains a paucity of research exploring the specific mechanisms connecting these variables and cyberbullying perpetration. Specifically, the predictive role of these variables in cyberbullying perpetration needs further exploration. Also, by scrutinizing the interrelationships among potential predictors and investigating their pathways to cyberbullying, it is possible to develop a more comprehensive analytical framework for understanding cyberbullying perpetration. The present study was designed to address these considerations.

**Fig. 1:**
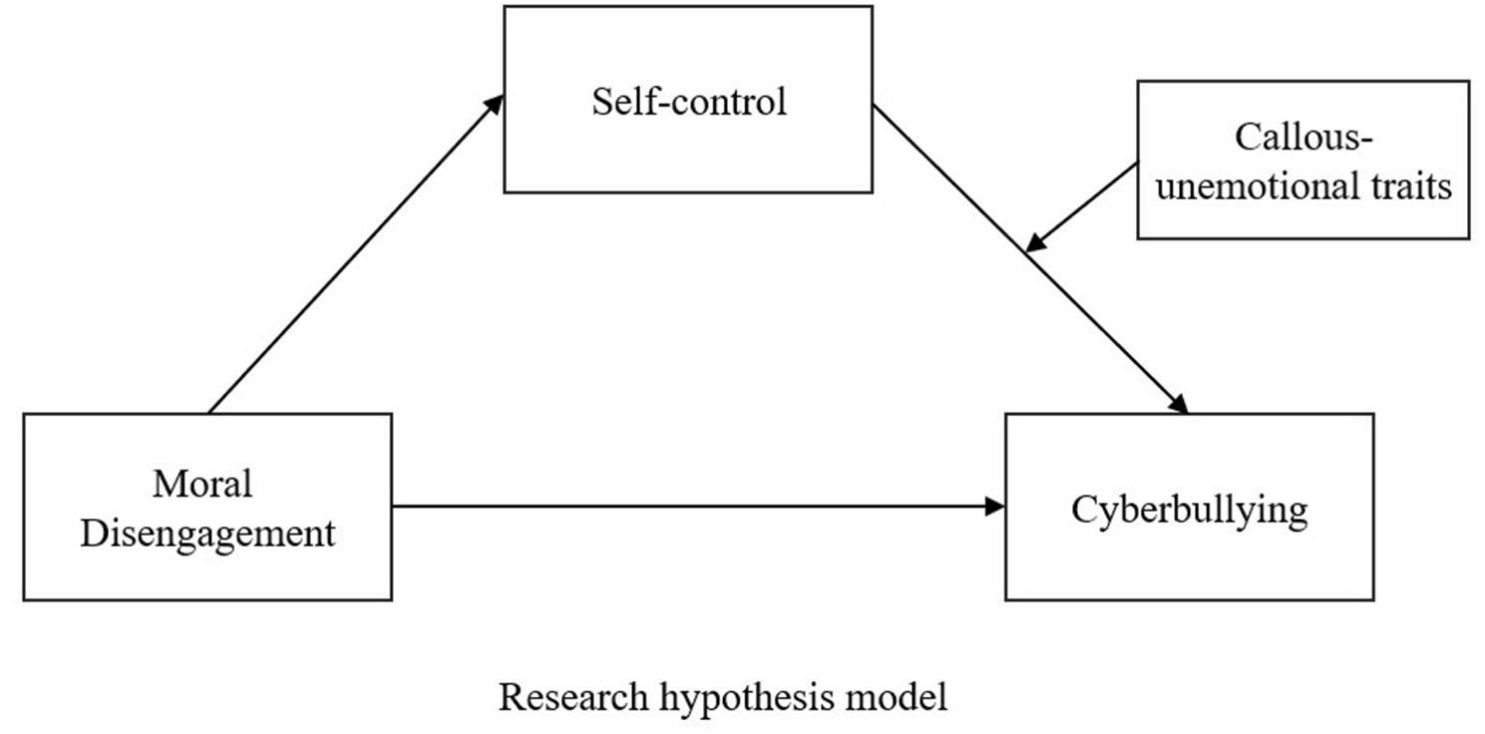
Proposed theoretical model

The hypothesis of current study was thus designed to shed light on a mechanism by which moral disengagement predicts cyberbullying perpetration, together with the role of self-control and callous-unemotional traits. Specifically, we investigated whether self-control mediates the relation between moral disengagement and cyberbullying perpetration and, if so, the extent to which individual differences in callous-unemotional traits might moderate the indirect path. As shown in Fig. 1, we propose the following hypotheses as part of our theoretical model:

#### H1

Moral disengagement is positively correlated with cyberbullying perpetration.

#### H2

Self-control mediates the relation between moral disengagement and cyberbullying perpetration, such that **(a)** higher levels of moral disengagement predict lower levels of self-control and **(b)** lower self-control predicts higher levels of cyberbullying.

#### H3

Moral disengagement and self-control jointly correlate with cyberbullying perpetration, which will vary by callous-unemotional traits. Specifically, the relation between self-control and cyberbullying is moderated by callous-unemotional traits.

The three hypotheses depict a moderated mediation model in which self-control serves as a mediator between moral disengagement and cyberbullying, and callous-unemotional traits function as a moderator of the relationship between self-control and cyberbullying perpetration.

## Methods

An a priori power analysis revealed that to test a simple mediation model with an anticipated small effect size for the view between moral disengagement to self-control and between self-control to cyberbullying indicated a minimum of approximately 560 participants would be needed to test for mediation using the parametric bootstrap procedure for calculating the standard error of the indirect effect [48].

In order to adequately power a test of moderated mediation, we anticipated needing a minimum sample size of 500 participants (using bootstrapped procedure to estimate SE) for potential moderated mediation given a small effect size [49].

Thus, to ensure adequate power across multiple hypotheses, we aimed to collect approximately 800 participants. A sample of 860 Chinese internet users 18 years of age or older completed an online questionnaire through wjx.com, an online survey platform. Before data collection, this study has been reviewed and approved by the Ethics Committee of Renmin University of China (Approval number 21–062; Approval date 3/15/2021). We shared the web link and invited eligible individuals to complete the questionnaire by reaching out to them on social media platforms. A cover letter detailing what research participation will entail and the potential benefits and risks of participating is included on the first page of the online survey. The completion of each questionnaire takes approximately 10–15 min, and participants will receive a cash reward upon finishing. After eliminating the careless and irresponsible response (n = 38) and answer time lower than 60 s (n = 1), a total of 818 valid questionnaires were included in the final sample.

*Demographic variables.* Participants indicated which category they belonged to 1 = *18–24 years old*, 2 = *25–29 years old*, 3 = *30 years old and above*. The highest level of education was measured on a 5-point scale with the following options: 1 = *Middle school and lower*, 2 = *High school*, 3 = *Some college*, 4 = *Bachelor*, 5 = *Master and above*. The frequency of internet use was measured using a 4-point scale with the following options: 1 = *Barely not* to 4 = *Frequent.* Finally, participants indicated whether their birthplace was rural or urban: 1 = *Rural*, 2 = *Urban*.

*Cyberbullying.* To measure cyberbullying, we used 10 questions (Cronbach’s α = 0.96) adapted from the Internet Excessive Behavior Subscale of the Adolescent Internet Deviation Behavior Questionnaire [50]. Sample items included “When there’s a conflict with others on the internet, I will send them offensive words, abbreviations or symbols”; “I will mock or ridicule others on the internet”; and “When I see someone bullying others I will participate in the bullying.” The items demonstrated excellent reliability, with a Cronbach’s alpha of 0.96. The results from all items were aggregated, and an arithmetic mean was computed, serving as a singular indicator for the entire scale. Higher scores denote a greater degree of cyberbullying perpetration.

*Moral disengagement.* To measure moral disengagement, we adapted items from the 20-item scale originally developed by Bandura [11] comprising four factors: moral justification, advantageous comparison, attribution of blame, and displacement of responsibility. Sample questions included “Damaging some property is no big deal when you consider that others are beating people up”, “ Stealing some money is not too serious compared to those who steal a lot of money.” Responses were measured on a 5-point scale from 1 = *Never* to 5 = *Always*. The items demonstrated excellent reliability, with a Cronbach’s alpha of 0.94. The results from all items were aggregated, and an arithmetic mean was computed, serving as a singular indicator for the entire scale. Higher scores reflecting a higher degree of moral disengagement.

*Self-control.* We used the 19-item Self-Control Scale by Tangney and colleague [51], which assesses resistance to temptation, healthy habits, temperance of enjoyment, impulse control, and focused work. Sample items include “I am good at resisting temptation” and “I have a hard time breaking bad habits” (reverse-scored). Responses were measured on a 5-point scale from 1 = *completely non-conforming* to 5 = *completely conforming*. The items demonstrated sufficient reliability, with a Cronbach’s alpha of 0.83. The results from all items were aggregated, and an arithmetic mean was computed, serving as a singular indicator for the entire scale. Higher values indicate stronger self-control.

*Callous-Unemotional Traits.* We used the Callous-Unemotional Traits Scale [52], which measures three dimensions of callous-emotional traits: coldness, numbness, and callousness. This questionnaire contains 21 items (e.g., “The feelings of others are not important to me.”) measured on a 4-point scale from 1 = *completely inconsistent* to 4 = *completely conforming*. The items demonstrated sufficient reliability, with a Cronbach’s alpha of .80. The results from all items were aggregated, and an arithmetic mean was computed, serving as a singular indicator for the entire scale. Higher scores indicate a higher degree of callous-unemotional traits.

We adapted the scales for measuring moral disengagement, self-control, callous-unemotional traits, and cyberbullying from the original versions—which were written in English—by translating them to Chinese. We enlisted experts to review the translations, enhancing readability in Chinese and clarifying ambiguous phrases to ensure that the meaning of items remained consistent before and after translation. We tested Cronbach’s alpha and found that all the adapted scales displayed sufficient reliability (above 0.80). The Chinese versions of these measures are available upon request from the lead author.

## Results

### Descriptive result

We employed Harman’s single factor test to conduct an exploratory factor analysis of all questionnaire items, excluding demographic variables. The total variance explained by the single factor amounted to 31.65%, which falls below the standard threshold of 50%. Consequently, this rules out the potential presence of common method bias. We conducted statistical analyses using SPSS 26 and employed the PROCESS macro [53] to examine mediation effects as well as moderated mediation in our study.

Table 1 shows the means, standard deviations, and bivariate correlations for age, education level, frequency of internet use, and the key study variables (i.e., moral disengagement, self-control, cyberbullying, callous-unemotional traits). Consistent with H1, moral disengagement was significantly positively correlated with cyberbullying. In line with H2, self-control was significantly negatively correlated with moral disengagement (2a) and cyberbullying (2b). At the bivariate level, callous-unemotional traits were significantly positively correlated with moral disengagement and cyberbullying and significantly negatively correlated with self-control.

**Table 1:** Descriptives and Bivariate Correlations for Major Study Variables

	*M*	*SD*	1	2	3	4	5	6
Age (by age group)	2.01	0.79	-					
Education	3.6	1.01	0.61	-				
Frequency of Internet Use	3.18	1	.11**	0.40**	-			
Moral Disengagement	2.63	0.84	− 0.13**	− 0.15**	− 0.42**	-		
Self-Control	3.09	0.55	0.17**	0.44	0.25**	− 0.68**	-	
Cyberbullying	2.13	1.05	− 0.16**	− 0.27**	− 0.57**	0.74**	− 0.60**	-
Callous Unemotional Traits	2.19	0.39	− 0.22**	− 0.31**	− 0.55**	0.47**	− 0.38**	0.59**

**p < .01, *p < .05

Additionally, to further control the impact of gender differences and socioeconomic status on cyberbullying perpetration and consequently mitigate the influence of these individual factors on the overall study, an independent samples *t*-test were performed to investigate potential gender (men vs. women) and birthplace (urban vs. rural, a common indicator of SES for Chinese samples ) differences in the key study variables (as shown in Table 2). Men were significantly higher in moral disengagement, *t*(816) = 4.10, *p* < .01) cyberbullying, *t*(816) = 5.27, *p* < .01) and callous-unemotional traits, *t*(816) = 5.52, *p* < .01) and significantly lower in self-control than women, *t*(816) = 2.95, *p* < .01). Urban participants were significantly higher in moral disengagement, *t*(816) = -2.21, *p* < .05, and lower in self-control than rural participants, *t*(816) = 2.13, *p* < .05.

**Table 2:** Descriptive Statistics by Gender & Birthplace

	Male (*n* = 416)		Female (*n* = 402)		Rural (*n* = 252)		Urban (*n* = 266)	
	*M*	*SD*	*M*	*SD*	*M*	*SD*	*M*	*SD*
Moral Disengagement	2.74	0.86	2.51	0.81	2.53	0.84	2.67	0.84
Self-Control	3.03	0.55	3.15	0.54	3.16	0.54	3.07	0.55
Cyberbullying	2.13	1.05	1.93	1.03	2.07	1.07	2.15	1.05
Callous Unemotional Traits	2.27	0.36	2.12	0.4	2.22	0.39	2.18	0.38

### Mediation analysis result

**Fig. 2:**
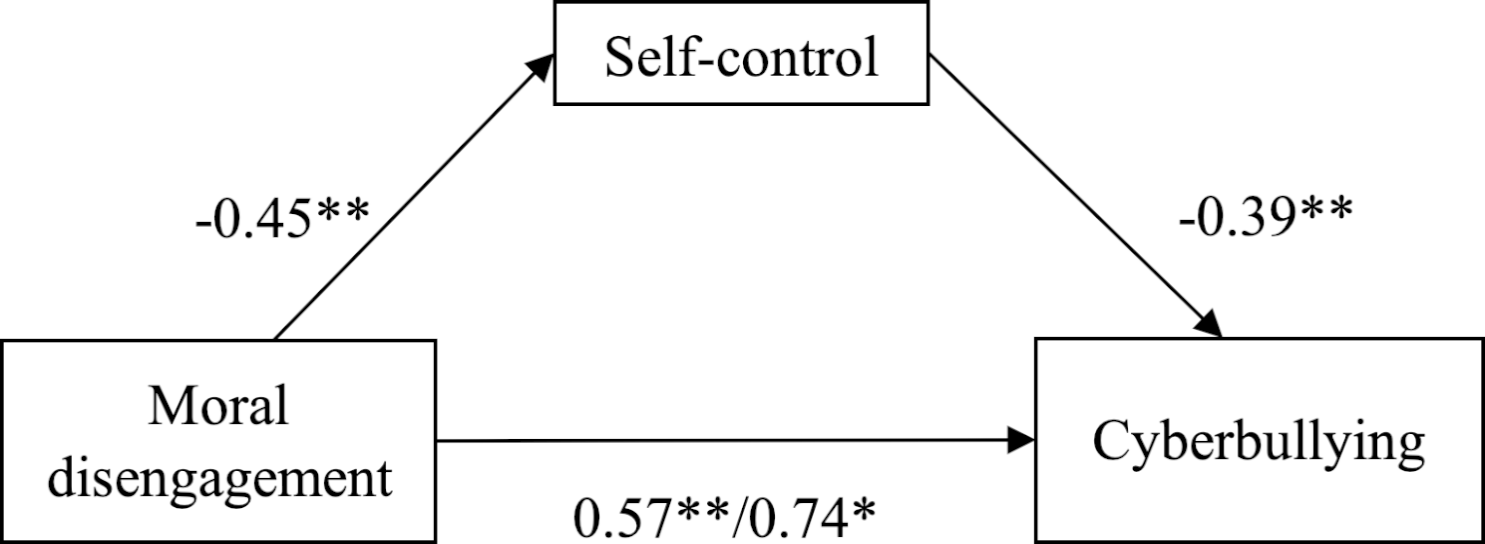
The mediation of moral disengagement and cyberbullying through self-control

To investigate the extent to which self-control mediates the relation between moral disengagement and cyberbullying, we tested a mediation model and included age, educational level, and internet usage as covariates in order to control for individual factors and exogenous variables that may impact our study. We employed a bootstrapping procedure (50,000 samples) to calculate the confidence interval of the indirect effect. As predicted, the effect of moral disengagement significantly predicted the value of self-control (*a* = -0.45, *SE* = 0.02, *t*(812) = -24.65, *p* = .001, 95% CI = -0.49, -0.42). Additionally, the effect of self-control on cyberbullying perpetration, controlling for moral disengagement, was significant (*b* = -0.39, *SE* = 0.02, *t*(812) = -7.17, *p* = .001, 95% CI = -0.50, -0.28). Also, self-control mediated the relation between moral disengagement and cyberbullying, with the significance of the indirect effect confirmed by a 95% confidence interval that did not include zero (*ab* = 0.18, *SE* = 0.03, 95% CI = 0.13, 0.24). Notably, when taking the mediation pathway into account, the total effect of moral disengagement on cyberbullying is significant (*c* = 0.75, *SE* = 0.03, *t*(812) = 25.19, *p* = .001.), as well as the direct effect (*c*’ = 0.57, *SE* = 0.04, *t*(812) = 14.95, *p* = .001.) Brief results as shown in Fig. 2.

### Test of moderated mediation result

We used the PROCESS macro (Model 14) to investigate whether the strength of the indirect effect of moral disengagement as a predictor of cyberbullying (through self-control) was moderated by participants’ level of callous-unemotional traits. We hypothesized that callous-unemotional traits would specifically moderate the relation between self-control and cyberbullying. Age, education, and internet use were once again included as covariates. Across all levels of callous-unemotional traits, self-control significantly mediated the relation between moral disengagement and cyberbullying (-1 *SD* callous-unemotional traits: *ab* = 0.10, *SE* = 0.03, 95% *CI* = 0.05, 0.15; mean callous-unemotional traits: *ab* = 0.19, *SE* = 0.03, 95% *CI* = 0.13, 0.26; +1 *SD* callous-unemotional traits: *ab* = 0.29, *SE* = 0.05, 95% *CI* = 0.20, 0.38). Crucially, however, the indirect effect was significantly stronger for participants who were relatively higher in callous-unemotional traits, as confirmed by the significant index of moderated mediation (index = 0.25, *SE* = 0.05, 95% *CI* = 0.15, 0.35).

**Fig. 3:**
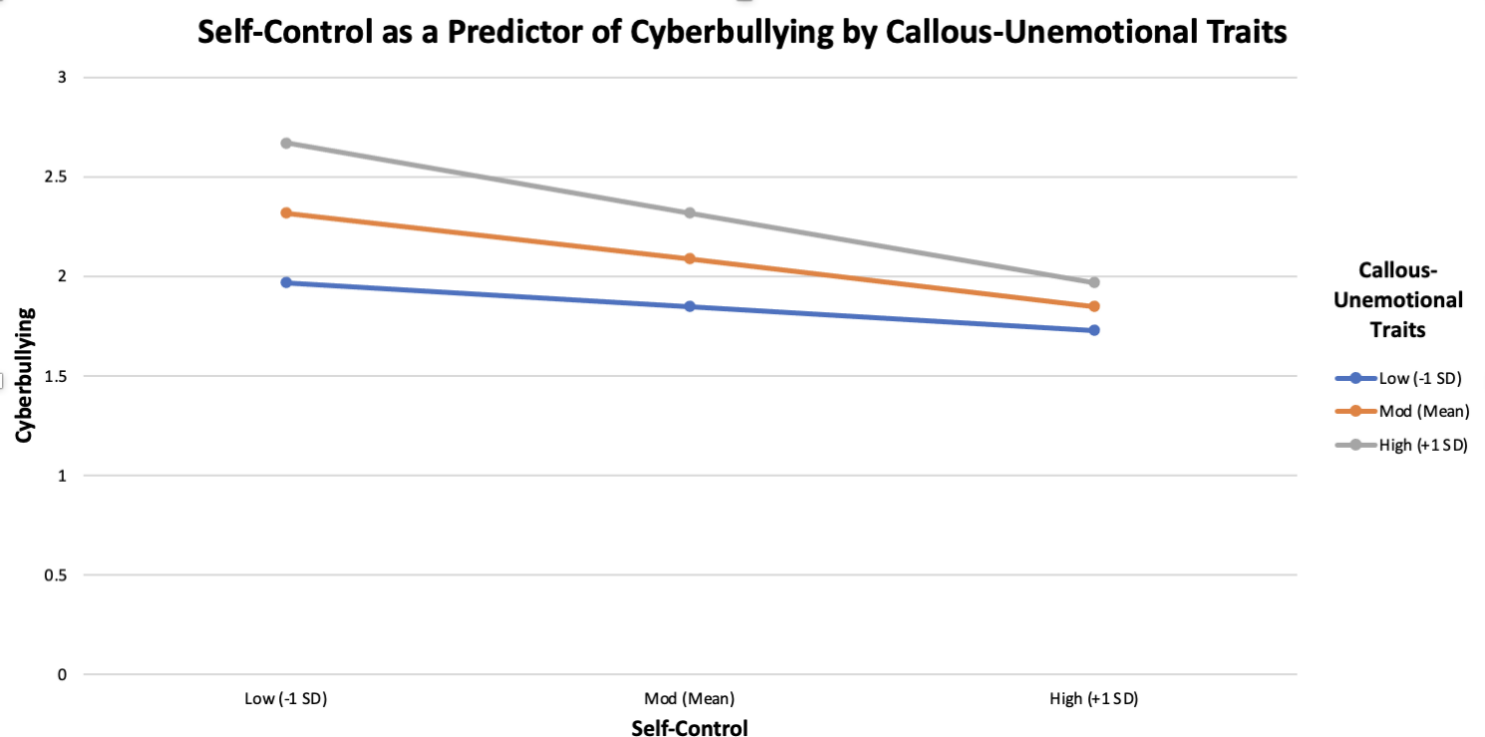
Moderation of self-control and cyberbullying by callous-unemotional traits

To further probe the moderation effect, a model was tested with self-control (mean-centered) as the focal predictor of cyberbullying, callous-unemotional traits (mean-centered) as the moderator, and cyberbullying as the dependent variable, with age, education, and internet use as covariates (as shown in Fig. 3). Conditional main effects for self-control and cyberbullying emerged, such that participants lower in self-control, *b* = -0.42, *SE* = 0.06, *t*(812) = -7.62, *p* < .001, and participants higher in callous-unemotional traits, *b* = 0.61, *SE* = 0.08, *t*(812) = 8.03, *p* < .001, reported higher levels cyberbullying. These effects were, however, qualified by a significant interaction between self-control and callous-unemotional traits, *b* = -0.54, *SE* = 0.12, *t*(812) = -4.86, *p* < .001, Δ*R*^2^ = 0.01. Simple slopes analyses revealed that, whereas higher self-control predicted lower levels of cyberbullying across all levels of callous-unemotional traits (-1 *SD* callous-unemotional traits: *b* = -0.21, *SE* = 0.06, *t*(812) = -3.52, *p* < .001; mean callous-unemotional traits: *b* = -0.42, *SE* = 0.06, *t*(812) = -7.62, *p* < .001; +1 *SD* callous-unemotional traits: *b* = -0.63, *SE* = 0.09, *t*(812) = -8.02, *p* < .001), this negative relation was significantly stronger for those with relatively higher levels of callous-unemotional traits. In other words, low self-control was an especially strong predictor of cyberbullying among participants with greater callous-unemotional traits.

## Discussion

Although previous studies have identified a connection between moral disengagement and cyberbullying perpetration [20, 54], there is currently insufficient research to effectively elucidate the mechanism through which this psychological characteristic exerts a substantial impact on cyberbullying. Also, previous research ignored the close ties between moral disengagement, self-control and callous-unemotional traits on predicting the aggressive behavior [37, 47], which makes it necessary to reconsider such links and how it influenced the predicting effect on cyberbullying. Building on prior research, this study proposes a potentially significant new mechanism by which moral disengagement is linked with cyberbullying: decreased self-control, which holds meaningful implications for our further understanding of this issue. Self-control mediates the relationship between moral disengagement and cyberbullying. Though moral disengagement positively predicts cyberbullying, this effect is attenuated by self-control. This attenuation is evident from the lower slope of interaction effect and the negative correlation between the two variables. These findings corroborate that the combined effect of moral disengagement and self-control on cyberbullying is valid, aligning with previous research on other forms of aggressive behavior [36–38].

Additionally, the present research revealed that the relation between self-control and cyberbullying depends on one’s degree of callous-unemotional traits. Our findings suggest that individuals with relatively higher levels of callous-unemotional traits are particularly likely to harm others online and are thus consistent with previous work linking callous-unemotional traits with greater impulsive and aggressive behaviors [43, 55]. This aspect is often easily overlooked, as people tend to associate callous-unemotional traits with physical aggression in real-life settings, while neglecting that in online environments and situations of anonymity and indirect contact [56], some dark traits and negative traits in personality may be more readily expressed due to the lack of substantial constraints and lower costs of infringement [57]. Consequently, callous-unemotional traits might manifest more prominently and extremely on the internet, which leads to an increase in the likelihood of aggression. The findings of this study on callous-unemotional traits contribute to further explaining the mechanisms through which negative personality traits affect cyberbullying perpetration.

Yet, we also believe this research has practical significance. This study tested a model of moral disengagement, self-control, callous-unemotional traits, and cyberbullying that can provide a theoretical foundation for potential intervention strategies targeting cyberbullying, help inform the efforts of educators and policymakers to prevent or reduce cyberbullying. For example, the present research underscores the role of self-control in cyberbullying perpetration, and there are well-established precedents for external interventions targeting self-control capabilities [58, 59]. In light of this, school (or university) administrators might develop cyberbullying interventions aimed at increasing students’ state or trait levels of self-control or increasing students’ awareness of their levels of self-control while engaging in online interactions. A complementary approach may be to tailor cyberbullying interventions based on individuals’ levels of moral disengagement and/or callous-unemotional traits so that individuals at greater risk for cyberbullying others receive stronger interventions. These potential interventional applications offer a promising future research direction, such as conducting experiments to verify whether interventions targeting self-control could help reduce the likelihood of cyberbullying perpetration. Moreover, some studies have discussed the possibility of intervening in moral disengagement through educational means [60, 61], which could also become a future research direction. By directly addressing the psychological mechanisms underlying cyberbullying perpetration, we can potentially devise more effective prevention strategies. This would enable governments and administrators to implement a broader range of interventions to combat cyberbullying, such as nationwide projects initiated by governments [62]. Of course, further understanding the personality traits that trigger cyberbullying is also essential. The present study proposes a possible framework for the influence of moral disengagement, self-control, and callous-unemotional traits on cyberbullying, but does not rule out the possibility that other personality traits might also play a role within this framework.

Furthermore, this research shows certain cross-cultural validity. Through our sample was collected from monocultural background, the research measurement we used in this research has proved cross-cultural validity. There was previous research tested the self-control scale [63], moral disengagement scale [64] and callous-unemotional traits scale [65] showed great cross-cultural validity, which ensured certain cross-cultural effectiveness and comparability of this research.

## Limitations

There are, however, some noteworthy limitations of this study. First, the translation of the measures from English to Chinese may have reduced the validity of some questions in the scale. Due to the subpar quality of the existing Chinese versions of the scales, we opted for direct translation. Although we made efforts to maintain the reliability and validity of the scales post-translation, which included engaging experts for proofreading, potential alterations, or loss of meaning during translation might still occur, potentially undermining the validity of the measurement. Secondly, there were measurement considerations. During the research design phase, we contemplated various methodologies including task-based and experimental methods. However, given data availability, previous research usage (particularly with moral disengagement), and the feasibility and convenience when working with large samples, we opted to employ scales in this research. Nevertheless, the use of scales introduced some problems. Given the sole reliance on self-report instruments, there is a potential risk of participants not responding honestly, due to social desirability biases or memory distortions. In particular, when inquired about negative past experiences or immoral behaviors, such as cyberbullying, participants might subconsciously portray themselves in a more positive light, leading to inadvertent misrepresentation in their responses. Third, the monocultural background of the participants underscores the need for future cross-cultural replications of the present study. Although there are a handful of cross-cultural studies on the topic of cyberbullying [35], the majority of existing research utilizes monocultural samples, making it challenging to ascertain the cross-cultural applicability of the findings. To address these limitations, it is necessary to conduct further research on this issue. For example, incorporating a cross-cultural analytical framework and comparing whether the manifestations and impacts of moral disengagement are the same for people under different cultures, designing behavioral experiments to better analyze people’s tendencies to engage in cyberbullying, and further improving the accuracy of existing analytical tools and scales, among other approaches.

## Conclusion

In a cross-sectional survey of participants in China, moral disengagement predicted higher levels of cyberbullying. Consistent with hypotheses, individual differences in self-control mediated this relation. Furthermore, evidence of moderated mediation emerged, such that the link between self-control and cyberbullying was moderated by callous-unemotional traits. In other words, the magnitude of the indirect effect varied as a function of the degree of callous-unemotional traits, with a significantly stronger indirect effect among individuals with relatively higher levels of callous-unemotional traits.

The results proposed in this study contributes to a deeper understanding of the underlying mechanisms in the formation of cyberbullying behavior, clarifies the relationships among potential predicting variables, and provides a theoretical foundation and potential avenues for future interventions aimed at addressing cyberbullying behavior.

## Data Availability

The datasets generated during and/or analyzed during the current study are available from the corresponding author on reasonable request.
